# Speech-induced atrial tachycardia: A narrative review of putative mechanisms implicating the autonomic nervous system

**DOI:** 10.1016/j.hroo.2023.07.006

**Published:** 2023-07-20

**Authors:** Gabriel M.Pajares Hurtado, John-Ross D. Clarke, Andre Zimerman, Timothy Maher, Liliana Tavares, Andre d’Avila

**Affiliations:** ∗ Cardiovascular Division, Department of Medicine, Beth Israel Deaconess Medical Center, Harvard Medical School, Boston, Massachusetts; † TIMI Study Group, Brigham and Women’s Hospital, Harvard Medical School, Boston, Massachusetts; ‡ Harvard-Thorndike Electrophysiology Institute, Cardiovascular Division, Department of Medicine, Beth Israel Deaconess Medical Center, Harvard Medical School, Boston, Massachusetts; § Department of Medicine, MetroWest Medical Center, Tufts School of Medicine, Framingham, Massachusetts

**Keywords:** Atrial tachycardia, Speech production, Autonomic nervous system, Central nervous system, Mechanisms of arrhythmia

## Abstract

Despite being uncommon, speech-induced atrial tachycardias carry significant morbidity and affect predominantly healthy individuals. Little is known about their mechanism, treatment, and prognosis. In this review, we seek to identify the underlying connections and pathophysiology between speech and arrhythmias while providing an informed approach to evaluation and management.

**Key Findings**

▪ Speech-induced atrial tachycardias are rare, quality-of-life–limiting arrhythmias that predominantly affect previously healthy individuals without structural heart disease.
▪ The mechanism of speech-induced atrial tachycardias likely involves the autonomic nervous system, thus implicating aberrant stimulation through extrinsic and intrinsic cardiac autonomic structures.
▪ Evidence is limited with regard to treatment strategies and range from beta-blockade to catheter ablation, with the former being effective for transient episodes of speech-induced tachycardias, and the latter showing a marked predilection for ablation targets in areas associated with intrinsic cardiac structures.

## Introduction

The autonomic nervous system (ANS) plays a complex role in the modulation of cardiac electrophysiology and has been shown to potentiate various cardiac arrhythmias.^1^ Atrial tachycardias (ATs) comprise 5%–15% of supraventricular tachycardias in patients referred for catheter ablation. They are broadly characterized as macroreentrant or focal, the latter originating from a localized area in the atria.^2^ Focal ATs can arise from automaticity, triggered activity, or microreentrant mechanisms. Although focal AT typically is considered a benign arrhythmia, patients with the condition may present with symptoms related to increased heart rate, including palpitations, lightheadedness, and shortness of breath.

A recent 2021 case report described a previously healthy 58-year-old man who presented to the emergency department with a 1-month history of dizziness and palpitations that occurred solely while the patient was speaking.^3^ Cardiac rhythm monitoring showed a speech-induced AT. Speech-induced ATs are a unique yet poorly characterized phenomenon. Since the first case of a “pharyngeal arrhythmia” published in 1926,^4^ subsequent case reports have provided distinctive insights into speech-induced tachycardias, including the potential role of the ANS in their arrhythmogenesis. However, current literature does not offer a unified description of the underlying pathophysiological mechanism or a well-defined approach to their evaluation and management.^3^^,^^5^, ^6^, ^7^, ^8^, ^9^, ^10^, ^11^, ^12^, ^13^, ^14^

Because of the complexity of the systems involved in speech production and their neuroanatomic associations with arrhythmogenesis, a systematic analysis of previous reports may improve our understanding and treatment of these arrhythmias. Furthermore, insight into their mechanism holds potential for advancing our understanding of neural control of the heart. In this article, we review the literature on speech-induced tachycardias, propose possible mechanisms, describe their clinical features, and suggest a pathway to diagnosis and treatment.

### Case reports and clinical features

Initial presentations were similar in the cases reviewed. Patients were an average age of 59 years, and most (7/11) were male (Table 1). All patients had as their chief complaint palpitations with the onset of speech; 1 patient also experienced palpitations with coughing or swallowing.^6^ Most patients obtained immediate relief of symptoms with the cessation of speech. Notably, the patients were otherwise healthy. No cardiovascular history or use of daily medications was reported, except for 1 case with known atrial fibrillation (AF).^8^ No significant event occurred before symptom onset. Furthermore, there were no reported cases of structural heart disease as assessed by echocardiography and/or computed tomography.

**Table 1:** Published cases of speech-induced atrial tachycardias

Case	Age (y)/sex	Presentation	Structural abnormality	Location of arrhythmia	Treatment	Outcome
Kan et al (1981)	54/M	SVT triggered by speech, conversion to NSR with cessation of speech	None	Unknown	Response to digoxin and procainamide or digoxin and disopyramide	Unknown follow-up
Omori et al (1984)	57/M	Tachyarrhythmia triggered by speech, cough, swallowing	None	Unidentified atrial origin	Improvement with atropine	Unknown follow-up
Gallagher et al (2007)	68/F	AF triggered by speech, conversion to NSR with cessation of speech	Unknown	Anterior, ostial LSPV	Refractory to propafenone, sotalol, and dofetilide RFA successful	No recurrence at 9 mo
Fan et al (2012)	63/M	AT triggered by speech, conversion to NSR with cessation of speech	None	Roof of left atrium	RFA successful	Unknown follow-up
Medina et al (2013)	46/M	AT triggered by speech and during mid-inspiration, conversion to NSR with cessation of speech	Unknown	RSPV, right anterior ganglionated plexus	Refractory to ablation of posteromedial SVC Balloon cryoablation successful	Unknown follow-up
Memon et al (2013)	63/M	AT triggered by speaking in radio-voice, conversion to NSR with cessation of radio-voice	None	Ostium of RSPV	Refractory to sotalol RFA successful	No recurrence at 12 mo
Zucchelli et al (2014)	34/F	Incessant AT triggered by speech	None	Junction between SVC and anteroseptal region of cranial RA	Refractory to propafenone, moderate response to metoprolol and flecainide RFA successful	No recurrence at 6 mo
Ueno et al (2014)	63/M	AT triggered by speaking, conversion to NSR with cessation of speech	None	Septal side of SVC	RFA successful	No recurrence at 12 mo
Khemka et al (2015)	78/F	AT triggered by speaking or whispering, symptoms ceased with silence	None	Mid–crista terminalis Concealed AV reentrant tachycardia s/p ablation	Refractory to metoprolol and sotalol. RFA successful	Unknown follow-up
Liu et al (2019)	70/F	AT triggered by speaking, symptoms ceased with silence	None	Left posterior–superior wall of the left atrium	RFA successful	No recurrence at 12 mo
Zimerman et al (2021)	58/M	PACs and sustained AT triggered by speech, conversion to NSR with cessation of speech	None	Unknown	Metoprolol successful	No recurrence at 3 y

Literature review was conducted using the PubMed database using the search terms autonomic nervous system, ganglionated plexi, vagal, neural mechanisms, atrial fibrillation, atrial tachycardia, arrhythmia, and ablation. Full-text articles published from 1981 to 2022 were included. Reference lists of comprehensive review articles were further examined for additional references.

AF = atrial fibrillation; AT = atrial tachycardia; AV = atrioventricular; F = female; LSPV = left superior pulmonary vein; M = male; NSR = normal sinus rhythm; PAC = premature atrial contraction; RA = right atrium; RFA = radiofrequency ablation; RSPV = right superior pulmonary vein; SVC = superior vena cava; s/p = status post; SVT = supraventricular tachycardia.

The hallmark electrocardiographic features of speech-induced AT include organized, irregular atrial activity at 150–250 bpm yielding a narrow-complex, long RP tachycardia triggered by the onset of speech. These broad electrocardiographic characteristics were described in all cases except for the 1 patient in whom AF was triggered by speaking.^7^ Termination of the tachyarrhythmia with return to normal sinus rhythm upon cessation of speech was described in most cases. P-wave morphology and length of PR interval varied depending on the location of the arrhythmogenic focus. In patients who underwent evaluation with electrophysiological study (8/11), the arrhythmogenic origin was identified and ablated without reported recurrence. There was predilection to areas susceptible to autonomic input in areas associated with ganglionated plexuses (GPs).

### The ANS and atrial tachyarrhythmias

The role of the ANS in modulation of cardiac electrophysiology and arrhythmogenesis has undergone extensive review because of great interest given both pathophysiological understanding and therapeutic potential.^1^^,^^15^^,^^16^ Described as a 3-level hierarchy, cardioneural connections are organized into higher cortical centers, brainstem, and spinal cord (level 1); intrathoracic extracardiac neurons (level 2); and all intrinsic cardiac neurons (level 3).^17^^,^^18^

**Figure 1:**
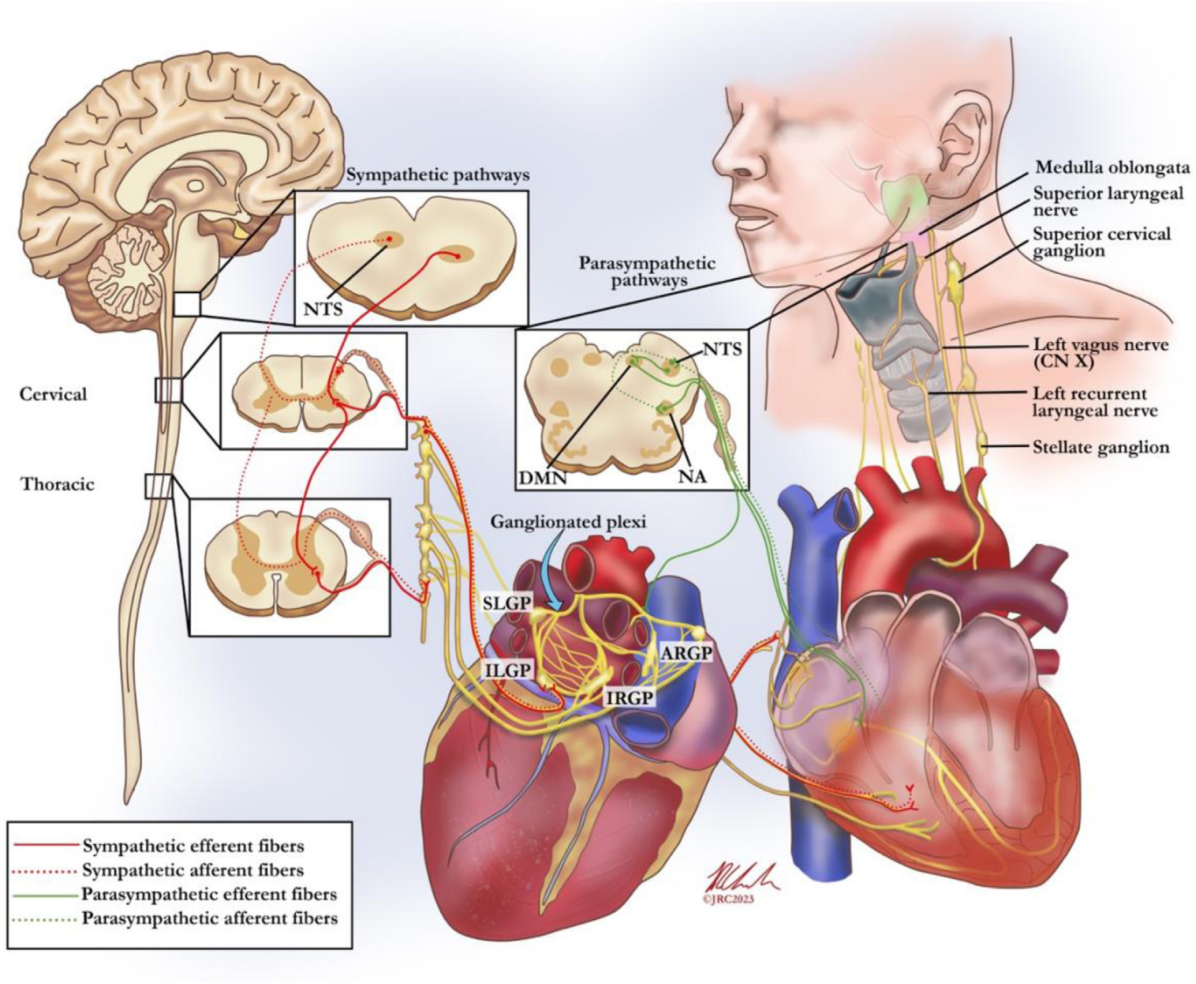
Neuroanatomy of speech production and the autonomic nervous system of the heart. ARGP = anterior right ganglionated plexus; CN = cranial nerve; DMN = dorsal motor nucleus; ILGP = inferior left ganglionated plexus; IRGP = inferior right ganglionated plexus; NA = nucleus ambiguus; NTS = nucleus tractus solitarius; SLGP = superior left ganglionated plexus.

Acknowledging that the extrinsic cardiac ANS ultimately is not dichotomous in its function or dysfunction, it generally consists of both sympathetic (superior cervical ganglia, stellate ganglia, and thoracic ganglia) and parasympathetic (originating at the nucleus ambiguus and carried almost entirely within the vagus nerve) components.^19^ Although vagal innervation is commonly associated with a purely parasympathetic response, the vagus has been shown to carry sympathetic nerve fibers, which can accelerate heart rate with low-output vagal stimulation.^20^ Serving as one of many integration centers for incoming sympathetic and parasympathetic information, the numerous GPs composing the intrinsic cardiac ANS function to modulate communication between extrinsic and the rest of the intrinsic cardiac ANS.^21^^,^^22^ GPs have been localized at particularly high densities within the atria and specifically around the pulmonary vein–left atrium junction, which contains both parasympathetic and sympathetic efferent, afferent, and local circuit neurons (Figure 1).^23^ Additionally located at the pulmonary vein–left atrium junction are afferent volume-sensing receptors that act to detect fluctuations in atrial stretching and mitigate appropriate chronotropic effects.^24^

Sympathetic signaling in the atria yields a surplus of calcium secondary to a spontaneous release of calcium from the sarcoplasmic reticulum, creating an environment of calcium handling dysfunction.^25^ Atrial parasympathetic activation augments spatial electrophysiological heterogeneity, reduces the atrial effective refractory period, and promotes early afterdepolarizations toward the end of phase 3 in the cardiac action potential.^26^, ^27^, ^28^ With simultaneous sympathetic and parasympathetic activation, the atrial tissue is particularly vulnerable to arrhythmogenesis given the combination of increased calcium availability and shortening of the action potential duration fostering an environment for late phase 3 early afterdepolarizations, which can induce triggered activity and AF.^29^^,^^30^ With coexisting adrenergic and cholinergic afferent, efferent, and local circuit innervation, GPs and other associated ANS structures (eg, volume-sensing receptors) are susceptible to producing arrhythmias, especially involving pulmonary vein myocytes, which possess a naturally abbreviated action potential duration.^31^ Nevertheless, the ultimate role of GPs in the genesis or maintenance of tachyarrhythmias, particularly AF, remains an area of active basic and translational scientific investigation, with the long-term effects of ablation vs modulation of these structures remaining elusive.^32^, ^33^, ^34^, ^35^

The relationship between AF and the ANS offers an arguably analogous model to that of speech-induced tachycardias and thus adds further insight into the complex interplay between autonomic function and tachyarrhythmias. The dictum of “AF begets AF” is supported by contemporary evidence, which suggests that afferent signals such as rapid stimulation of the atria, whether in the form of a sustained tachyarrhythmia such as AF or in the laboratory with rapid atrial pacing, lead to perturbations in the ANS itself, resulting in a vulnerable arrhythmogenic substrate.^27^^,^^34^^,^^36^^,^^37^ More recently, heart–brain interaction and its observed impairment during AF represented by attenuated heart-evoked potentials within the right insula has revealed evidence supporting the role of interoceptive neurotransmission as another pathway for AF-mediated central and ANS dysfunction.^38^^,^^39^ These findings reveal the multilimb (efferent, afferent and interoceptive) and multidirectional character of the relationship between atrial tachyarrhythmias and ANS dysfunction.

### Mechanism of speech-induced arrhythmias

Speech-induced tachyarrhythmias are supraventricular arrhythmias triggered by the onset of speech production with a propensity to terminate or wane with cessation of speaking. Various theories have been postulated regarding the mechanism of arrhythmogenesis, with most highlighting an association between cardiac and laryngeal autonomic connections mediated by the vagus nerve and its branches.

In the first published case of speech-induced tachycardia, Kan et al^5^ suggested that enhanced automaticity of an ectopic focus mediated by abnormal vagal nerve stimulation from vocal cord activation triggered the narrow-complex tachycardia; alternatively, it was also proposed that microreentry among the neighboring cells in the atrium could have played a role in initiation. Interestingly, the patient responded well to a combination therapy of digoxin and class Ia antiarrhythmic drugs (disopyramide, procainamide) despite the well-known vagotonic effects of digoxin and vagolytic effects of disopyramide (and, to a lesser extent, procainamide). In a 1984 report of a narrow-complex tachyarrhythmia triggered by speech, cough, and swallowing, Omori et al^6^ proposed that vagal stimulation played an important role, adding that vagal activation likely hyperpolarized the atrial resting membrane potential, depressed phase 4 depolarization, and shortened the outward potassium conduction, effectively shortening the action potential. It was observed that digoxin worsened the arrhythmia, and atropine, a vagolytic agent, seemed to improve symptoms, further supporting a mainly vagally mediated mechanism. Subsequent cases added to this theory, proposing that an anomalous efferent input from the recurrent laryngeal nerve was abnormally activating left atrial GP via the vagus.^3^^,^^8^ However, both Ueno et al^12^ and Khemka et al^13^ argued that the aberrant signal was of supramedullary origin, given that the speech-induced AT began immediately before speech initiation. The authors suggested that central modulation acting on the pharyngeal muscles via the vagus nerve also abnormally stimulated cooperative activation of sympathetic and parasympathetic tone to atrial tissue via GPs.^29^ Liu et al^14^ favored the supramedullary mechanism, yet they recognized that anatomic changes seen in speech production may also play a role.

Medina et al^9^ acknowledged that targeting the anterior right GP during catheter ablation of a speech- and breathing-induced AT resulted in cessation of the arrhythmia. Given that the AT described initiated with the first word during mid-inspiration and subsided between sentences, the authors postulated that stretch receptors in the great vessels near the anterior right GP may be susceptible to activation secondary to local stretch. Movements involving the diaphragm or trachea while a person is speaking or breathing could activate these stretch receptors, resulting in afferent signals to the medulla and a fast hypothalamic reflex within the nucleus of the solitary tract, with efferent parasympathetic discharge through the dorsal medulla to the cardiac ganglia.^15^^,^^40^ Memon et al^10^ also proposed 2 mechanisms in their case of speech-induced AT, attributing part of the pathophysiology to a “peripheral theory” in which anatomic shifts of the thorax during voice production may alter cardiovagal modulation, facilitating arrhythmia propagation in a predisposed substrate. Increases in left atrial pressure preceded episodes of AT in a case reported by Liu et al,^14^ who noted increases in rate and organization were noted emanating from superior pulmonary veins in AF during episodes of high atrial pressure.^41^ Mechanical stretch as a trigger for similar atrial tachyarrhythmias has been supported in other case reports^42^^,^^43^ and thus adds an ancillary mechanism to aberrant supramedullary activation when actions other than speech are involved as triggers. This phenomenon was not equally noted throughout all cases.

Electrophysiological studies performed during the reported cases showed a marked predilection for arrhythmogenic foci in areas susceptible to autonomic input associated with GPs, such as pulmonary vein ostia, right atrium–superior vena cava (SVC) junction, and SVC–aorta–right superior pulmonary vein junction. Of the 11 reported cases of speech-induced tachyarrhythmias, 5 were localized to the left atrium (4 involving the pulmonary veins) and underwent successful ablation^7^, ^8^, ^9^, ^10^^,^^14^; 3 cases involved the right atrium^11^, ^12^, ^13^; and in the remaining cases the site was unknown.^3^^,^^5^^,^^6^ Cases with foci involving the right atrium were near the SVC, which is associated with the “third fat pad,” a structure where most of the parasympathetic fibers converge between the SVC and aorta.^44^ These electroanatomic findings, both when involving pulmonary veins or when close to the SVC, provide further evidence of a vagally mediated mechanism.

Given these reports, we propose that cases of speech-induced AT are due to an aberrantly conducted supramedullary signal that initially was intended for speech production but ultimately triggered vagal activation of intrinsic cardiac autonomic structures (eg, GPs, atrial volume-stretch receptors). This in turn exposed susceptible atrial tissue to both sympathetic and parasympathetic inputs, resulting in increased calcium availability and shortened action potentials, respectively, and leading to triggered activity as the mechanism for arrhythmogenesis. Importantly, the role of both efferent and interoceptive limbs of the cardiac–autonomic relationship also could be implicated, as evidenced by the onset of speech-induced AT before production of speech in relation to atrial stretch receptors.

Of the 3 patients treated with medications alone, only 1 experienced resolution of their arrhythmia with the use of medical therapy alone, 1 of whom had reported follow-up.^3^ As Zimerman and D’Avila^3^ noted in their case report, the use of metoprolol resolved symptoms without recurrence at 3 years. Resolution without ablation in this patient suggests that, in some patients, aberrant vagal activation of intrinsic cardiac autonomic structures represents a transient phenomenon. Indeed, the range of length of symptoms before to presentation varied widely, with some patients experiencing symptoms 1–2 years before presentation.^7^^,^^13^ It is unlikely that aberrant activation would have been transient in these cases.

### Management

Despite varying approaches to management, no recurrence was noted in cases with reported follow-up periods (6/11), which ranged from 6 months to 3 years. Although a publication bias toward cases in which no further tachyarrhythmias resulted could be implicated, it is important to note the trend toward an overall favorable prognosis. Initial workup should follow that typical for evaluating palpitations as the chief complaint. If speech is identified as the trigger for symptoms, then rhythm monitoring (ambulatory monitoring or inpatient telemetry depending on location of presentation) is appropriate and should reveal a consistent and predictable relationship between the initiation of speech and development of a long RP tachycardia. Importantly, the correlation between cessation of speech and return to normal sinus rhythm should be investigated. Attempting other maneuvers such as Valsalva, mouthing words, deep breaths, coughing, or swallowing, which probe associations between potential vagally mediated outputs and the tachycardia, further complements a thorough evaluation. Further probing with direct neurological testing was not reported in the published case reports, yet data gained from electroencephalograms and functional or static neuroimaging while performance of the aforementioned maneuvers stands to better inform relationships between supramedullary signals and the observed arrhythmia. Identification of all stimuli improves the ability to elicit and reproduce the arrhythmia in an electrophysiological study if later indicated. Evaluation of structural heart disease should be prioritized; an echocardiogram with or without cross-sectional imaging of the chest often suffices.

Successful medical management alone was noted in only 3 reported cases, 1 of which described no recurrence at 3 years with metoprolol.^3^^,^^5^ The other 2 case reports described improvement with digoxin in combination with procainamide or disopyramide,^5^ and atropine^6^ without mention of a follow-up period or recurrence. Atrioventricular node blocking agents, mainly metoprolol, were attempted in 2 other cases without long-term success.^11^^,^^13^ Other than these medications, no other regimen seemed successful or well tolerated. Metoprolol did not worsen the arrhythmia in any case reported. Given their low-risk profile and potential transiency of the arrhythmia,^3^ beta-blockers are a safe and accessible option for the initial management of symptoms caused by speech-induced ATs. This is especially applicable in cases with continuing evaluation, patients with delayed access to an electrophysiological study, or those for whom invasive investigation is contraindicated. Other pharmacologic agents with less desirable side-effect profiles were not consistently efficacious and thus are poor first-line interventions.

Electrophysiological study with subsequent catheter ablation (either radiofrequency ablation or balloon cryoablation) was the most common and successful method of management (8/11), resulting in remission of the arrhythmia in all cases at their reported follow-up time (range 6 months to 3 years). Given consistent and immediate results, catheter ablation is reasonable in patients with recurrent symptomatic speech-induced AT, especially when pharmacologic therapy is not well tolerated or unsuccessful. Evidence of tachycardia-induced cardiomyopathy would be a clear indication for catheter ablation, regardless of symptoms. All but one of the catheter ablations were radiofrequency ablations. There are insufficient data to recommend one type of ablation over another.

## Conclusion

Speech-induced ATs are rare, quality-of-life–limiting arrhythmias. Their proposed mechanism likely involves an aberrantly conducted supramedullary signal that initially was intended for speech production but ultimately triggered vagal activation of intrinsic cardiac autonomic structures, leading to heightened autonomic atrial tone causing triggered activity yielding an AT while speaking. Importantly, cessation of speech stopped the tachyarrhythmia, suggesting a functional rather than anatomic or substrate-based abnormality. Although uncommon, the reviewed pathophysiology offers new insights into the complex interplay between ANS and cardiac pathophysiology.
